# Impact of fragmented habitats on reproductive success in dominant shrubs: natural selection on floral display and pollinator visitation

**DOI:** 10.3389/fpls.2025.1522871

**Published:** 2025-06-05

**Authors:** Min Chen, Zheng-jiao-yi Wang, Xu-jun Ma, Xue-yong Zhao

**Affiliations:** ^1^ State Key Laboratory of Ecological Safety and Sustainable Development in Arid Lands, Northwest Institute of Eco-Environment and Resources, Chinese Academy of Sciences, Lanzhou, China; ^2^ Urat Desert-grassland Research Station, Northwest Institute of Eco-Environment and Resources, Chinese Academy of Sciences, Lanzhou, China; ^3^ Key Laboratory of Stress Physiology and Ecology in Cold and Arid Region of Gansu Province, Lanzhou, China; ^4^ University of Chinese Academy of Sciences, Beijing, China; ^5^ Naiman Desertification Research Station, Northwest Institute of Eco-Environment and Resources, Chinese Academy of Sciences, Lanzhou, China

**Keywords:** fragmented habitat, floral display, pollen limitation, female fitness, pollinator visitation frequency

## Abstract

Flowering shrubs in fragmented habitats often experience pollen limitation, which can lead to strong natural selection on floral display and affect reproductive success. Effective management and protection of dominant shrubs in fragmented habitats require a thorough understanding of the factors driving plant reproduction. However, the impact of fragmented habitats on reproductive success through floral display and pollinator visitation has not been experimentally quantified. We examined pollen limitation by comparing female fitness between open-pollinated and hand-pollinated plants. We also assessed the impact of natural selection (β) on floral display by comparing natural selection gradients in plants from natural and fragmented habitats. Our results show that this species is pollen-limited, with supplemental pollen increasing female fitness by 32%. This species exhibits directional selection for more open flowers and longer keel petals. We found that the number of open flowers is the main factor influencing pollinator visitation frequency and female fitness, and that pollinator visitation positively affects female fitness. This study provides insights into how fragmented habitats influence linear selection gradients related to the number of open flowers. Furthermore, this study highlights that fragmented habitats significantly influence reproductive success, with floral display being a crucial factor to consider when designing conservation strategies for this population.

## Introduction

Habitat fragmentation is widely recognized as a major driver of biodiversity loss and the growing isolation of ecosystems (Newman et al., 2013). In evolutionary ecology, the floral display of plants influences reproductive success by affecting pollinator transfer efficiency. Habitat fragmentation can affect the plant flowering period and flower number, while human disturbance influences pollinator visitation. These factors result in variations in floral display that may ultimately impact pollination success (Zhong et al., 2019; Chen et al., 2022). Desert ecosystems typically feature sparse vegetation and are particularly sensitive to human disturbance (Villagra et al., 2009). Environmental changes can lead to variations in floral display (such as flower number and spatial arrangement) and pollinator activity (Chen et al., 2020). As human disturbance and environmental changes persist, alterations in habitat structure caused by degradation and desertification will diminish the reproductive success of many plant communities (Chen et al., 2022).

An insufficient supply of pollen can reduce pollination success and limit fecundity (Lennartsson, 2002; Knight et al., 2005). Pollen limitation refers to the insufficient quantity or quality of pollen transfer that impedes reproductive success (Gómez et al., 2010). Habitat fragmentation alters plant-pollinator mutualisms, as many flowering plants depend on pollinator services. The diversity and abundance of pollinators decrease in isolated habitats (Chen et al., 2020). Pollinator visitation can be limited by factors such as resource availability (e.g., rewards and the number of open flowers), which influence pollen transfer efficiency (Asikainen and Mutikainen, 2005). Inadequate pollen transfer is a key factor that limits pollinator visitation efficiency (Chen et al., 2022; Fernández et al., 2012). The quality and quantity of pollen that reaches the stigma are crucial factors influencing pollination success (Corbett, 2003; Harder and Aizen, 2010). There is substantial evidence of pollen limitation caused by insufficient pollinator visitation. Floral traits, particularly in flowering plants, show remarkable diversity in their impact on pollinator visitation efficiency (Ishii and Kadono, 2002).

Floral specialization plays a key role in plant-pollinator interactions, and the influence of natural selection on floral display warrants greater attention (Sletvold and Ågren, 2010). Floral display is positively correlated with pollinator visits, which in turn enhances female fitness (Sletvold et al., 2010; Sun et al., 2018). Pollen reception efficiency often influences natural selection on floral display and female reproductive success in many plant species (Knight et al., 2005; Ashman and Morgan, 2004). Pollinators are typically attracted to flowers with higher densities, as these flowers tend to offer more pollen and rewards (Ashman et al., 2004; Hadley and Betts, 2012). Additionally, pollinator visitation may decline in fragmented habitats, as environmental fragmentation and isolation can reduce pollinator foraging time and activity (Steffan-Dewenter et al., 2006). Pollinator-mediated selection for flower number may enhance pollination success (Sletvold and Ågren, 2010). A related study suggests that female reproductive success is positively correlated with plant height, flower number, and spur length in orchids (Gigord et al., 2001).

*Caragana korshinskii* Kom., a leguminous shrub, is a dominant species in desert steppe ecosystems, playing a crucial role in ecosystem stabilization and soil erosion prevention (Newman et al., 2013). As a nitrogen-fixing legume, it acts as a pioneer species for sand fixation in arid regions, enhancing soil fertility, promoting herbaceous vegetation growth, and bolstering ecosystem resilience (Yao et al., 2019). However, despite its ecological significance, *C. korshinskii* faces challenges related to habitat fragmentation and limited pollination, which may impact its long-term survival and regeneration. Predominantly pollinated by insects, the species’ reliance on biological pollination raises concerns about pollen limitation in fragmented habitats (Chen and Zhao, 2019). Given its ecological importance and dependence on pollinators, *C. korshinskii* serves as an ideal model for studying the effects of habitat fragmentation on pollen limitation in desert steppe ecosystems. However, studies directly quantifying the effect of natural selection on this species remain scarce, limiting the understanding of how floral display drives reproductive evolution in fragmented habitats. This study aimed to: 1) test the differences in pollen limitation intensity between open-pollinated and hand-pollinated plants, 2) quantify natural selection on floral display across different habitats, and 3) examine how fragmented habitats influence reproductive success via floral display, while comparing the relationships between pollinator visitation frequency and female fitness. Furthermore, this study provides strategies to improve reproductive success and conservation efforts for dominant shrub species.

## Materials and methods

### Study site

**Figure 1:**
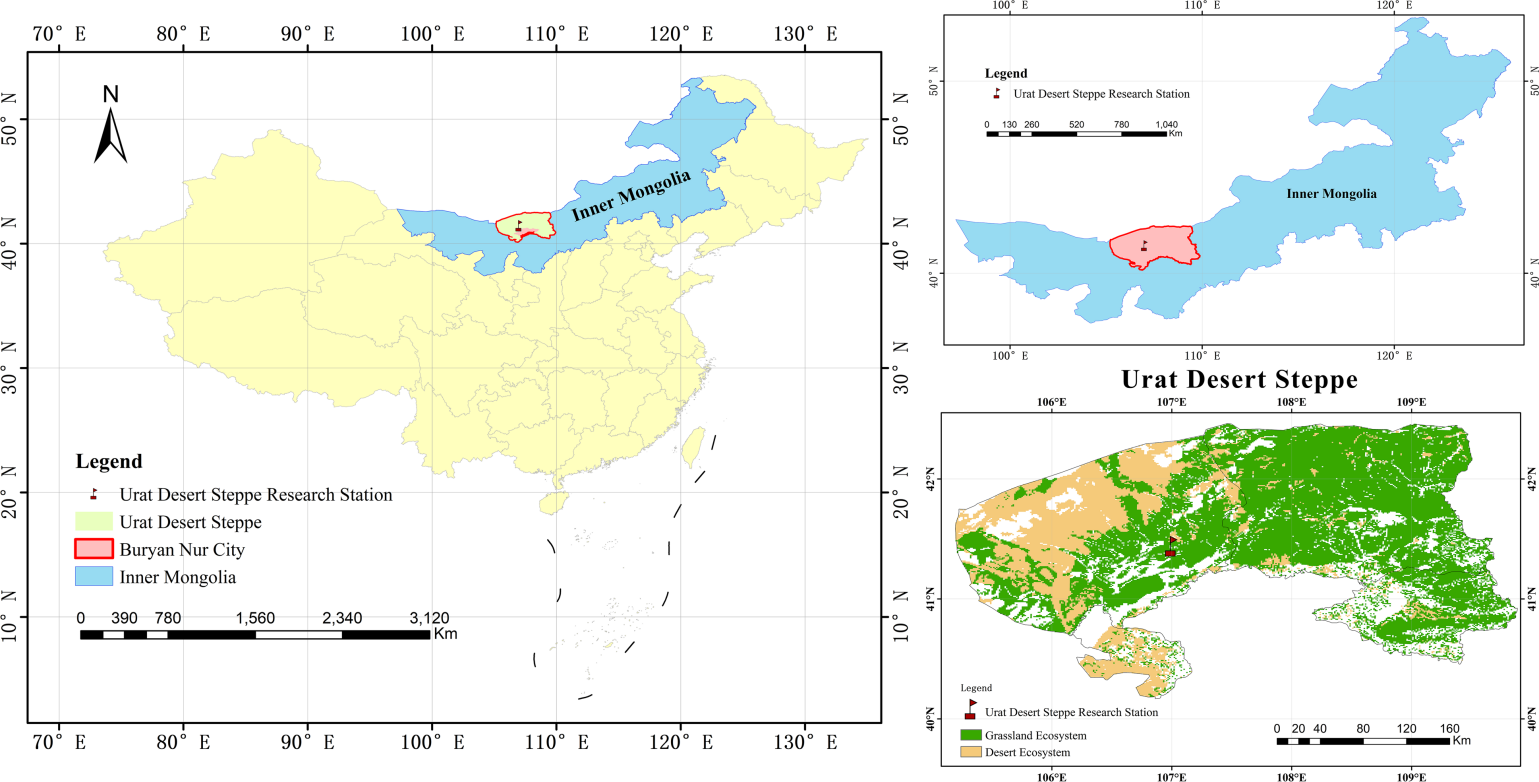
The study area is located in the Urat Desert steppe in Inner Mongolia Province, northwestern China.

This study was conducted in the Urat desert steppe (106°59’-107°05’E, 41°06’-41°25’N, **Figure 1**), located in the western part of Inner Mongolia Province, northwestern China. The region experiences a mean annual precipitation of approximately 153.9 millimeters, with the majority of precipitation concentrated between May and September, characteristic of a temperate steppe climate (BSk, Köppen Climate Classification; Guo et al., 2022). In this region, *Caragana korshinskii* Kom is the dominant shrub species (Wang et al., 2022).

### Study species

*C. korshinskii*, a widespread leguminous shrub in desert grasslands, is a vital component of these ecosystems. It significantly contributes to soil stabilization, nitrogen fixation, and the formation of the vegetation structure. This species is distributed across Inner Mongolia and Ningxia provinces in northern China. The flowering period of *C. korshinskii* typically lasts from May to July, while the fruiting period extends from July to August. Furthermore, *C. korshinskii*, primarily an outbreeding species with partial self-compatibility, relies on pollinators for effective pollen transfer despite some self-fertilization capacity (Chen and Zuo, 2019).

### Field experiment design

**Figure 2:**
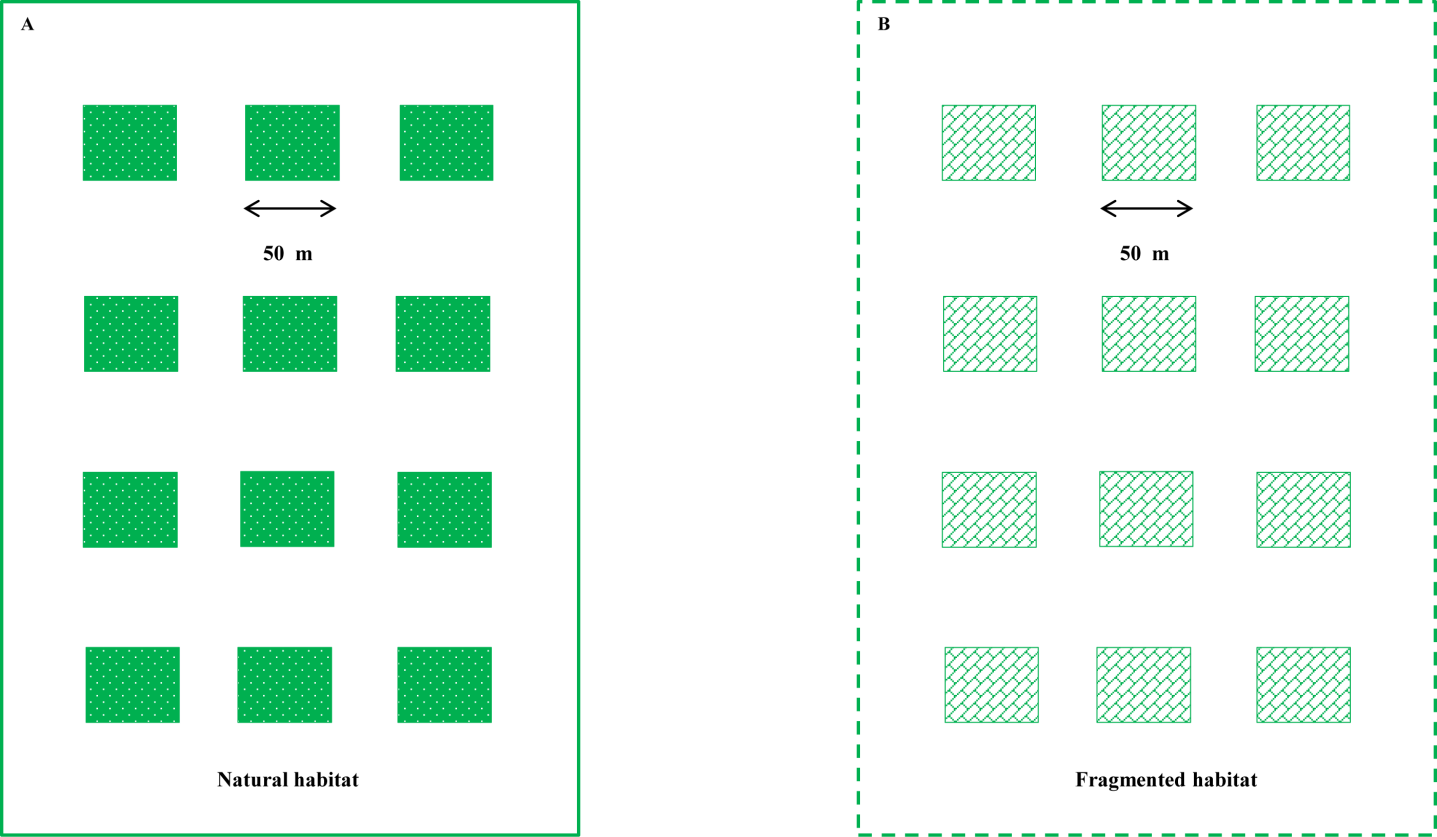
Experimental design for two treatments, natural habitat **(A)** and fragmented habitat **(B)**, from 2017 to 2023. The 24 selected plots (50 m × 50 m) were separated by mown vegetation (white area).

The experiment was conducted from May 2017 to September 2023. In 2017, age-matched *C. korshinskii* plants were selected for the study, with plant height standardized through regular cutting. Two experimental habitats were designed: 1) a natural habitat and 2) a fragmented habitat. Twelve plots were selected for each habitat, with habitats separated by 1 km to minimize pollinator interference. The fragmented habitat was located in a desert area with eroded topsoil. In the fragmented habitat, the twelve plots were distributed across cleared vegetated regions. The corresponding natural habitats were arranged similarly, with the same plant population (**Figure 2**). In 2023, experiments were conducted on floral display, natural selection, and pollinator visitation efficiency in both natural and fragmented habitats.

### Measured floral display

To assess floral display in both habitats, 144 plants with flowering buds were tagged across twelve natural and twelve fragmented plots (six plants per plot). In 2023, three inflorescences per tagged plant were marked, and digital calipers were used to measure keel petal length and corolla size (length and width) at the onset of flowering. In late May, tagged inflorescences with similar numbers of flower buds were selected. The number of open flowers was also recorded in June. Floral display indices of *C. korshinskii* were assessed as follows:


$${\text{I}}_{\text{FD}}=(\text{I}\times \text{AC})/100$$

where I_FD_ is the floral display index, I is the number of inflorescences, and AC is the area (m²) covered by plants in the natural and fragmented habitats. Seed production of the marked plants was evaluated during the seeding period, along with the relative female fitness of *C. korshinskii* in both habitats.

### Quantified natural selection

We quantified the intensity of natural directional selection (β) by analyzing floral displays and estimating the selection gradients in both natural and fragmented habitats (Sletvold and Ågren, 2010). We also evaluated floral displays, including the number of open flowers, corolla size, and keel petal length, and compared the selection gradients (β_N_ and β_F_) between natural and fragmented habitats.

### Pollen limitation

In 2023, we tested whether *C. korshinskii* plants experience pollen limitation by measuring mean female fitness in both open-pollinated and hand-pollinated plants. In each plot, we tagged 18 inflorescences (six plants, three per plant) with flowering buds for both open- and hand-pollinated treatments. During the flowering period, we conducted supplemental hand pollinations, and all the tagged flowers received supplemental pollen from untagged individuals. In late September, we counted mature seeds and seed masses in the laboratory. We calculated an index of pollen limitation using the mean fitness (number of intact seeds × seed mass) of open-pollinated (MF_C_) and hand-pollinated (MF_HP_) plants in different habitats:


$${\text{I}}_{\text{PL}}=1-({\text{MF}}_{\text{C}}/{\text{MF}}_{\text{HP}})$$

Positive values indicate greater mean fitness in the hand-pollinated (HP) treatment than in the open-pollinated (C) treatment, suggesting that *C. korshinskii* experiences pollen limitation. Negative or zero values indicate no pollen limitation.

### Effect of pollinator visitation on female fitness in fragmented habitats

We assessed the relationship between pollinator visitation frequency and relative female fitness to determine whether visitation frequency influences female fitness (Gómez et al., 2010). We tagged 144 plants (six per plot) in both natural and fragmented habitats based on floral display measurements. During the flowering period (June–July), we marked the number of open flowers on each tagged inflorescence and selected three inflorescences per labeled plant. We observed the labeled plants for five days during the flowering period. To prevent self-pollination, we removed the stamens from selected flowers. We recorded pollinator behavior and visit duration for the tagged flowers using an HD camera from 08:00 to 18:00. We captured the dominant pollinator with an insect net and identified it to the species level. A microscope was used to check whether the pollinators carried pollen grains. To prevent pollen contamination, we euthanized the captured insects in numbered glass bottles, each containing a single insect. In September, we recorded the number of open flowers (OF), pollinator visitation frequency (V_F_), and measured relative female fitness (R_FF_; individual fitness divided by mean fitness) for the tagged flowers.

We compared the relationships between OF and V_F_ and between V_F_ and R_FF_ to estimate how pollinator visitation affects female fitness (FF) in the visited flowers.

The pollinator visitation frequency is expressed as follows:


$${\text{V}}_{\text{F}}={\text{N}}_{\text{V}}/{\text{T}}_{\text{P}}$$

where NV is the number of pollinator visits to tagged plants and TP is the observation period (in days).

### Statistical analysis

We used Analysis of Variance (ANOVA) to assess the impact of fragmented habitats on OP, corolla size, and keel petal length. We applied the same models to test the impact of floral display on female fitness. When significant effects were detected, we performed *post-hoc* tests using Fisher’s least significant difference. We then performed linear regressions to measure selection.

Relative female fitness was used as the response variable. Floral displays (standardized by Z score: OF, corolla size, and keel petal length) were used as explanatory variables. Additionally, we quantified mean female fitness (number of seeds × seed mass) and calculated the standardized values for each floral display.

Initially, we evaluated female fitness and standardized floral displays in each habitat, incorporating quadratic terms (γii) to quantify nonlinear selection. However, quadratic gradients were statistically insignificant, so we report only linear gradients. We also assessed multicollinearity by inspecting variance inflation factors (VIFs) and found that the VIF for floral display was < 2.5, indicating that collinearity was not a significant issue.

The same models were applied to explore the relationships between OF and VF. Additionally, we conducted Spearman correlations. We performed linear regressions to assess the relationship between OP and V_F_. We also conducted linear regressions to explore the relationship between V_F_ and FF in different habitats. Finally, we applied piecewise structural equation modeling (SEM) to examine the direct and indirect pathways through which floral displays (OF, corolla size, and keel petal length) and pollinator visitation frequency influence female fitness in fragmented habitats (Hu et al., 2024). Model adequacy was assessed using Fisher’s C statistic in the “piecewiseSEM” package (R4.3.2) (Lefcheck, 2016). All statistical analyses were conducted using RStudio (R Core Team, 2023.09.1).

ANCOVA was applied to test whether different habitats affect linear selection gradients. We used relative fitness as the dependent variable. Additionally, standardized floral displays, habitat type (natural vs. fragmented), and the interaction between floral display and habitat were used as independent variables. To examine the effect of fragmented habitats, we quantified the estimated selection gradient for each floral display in different habitats. The analyses were performed using SPSS 24.0.

## Results

### Floral display

**Figure 3:**
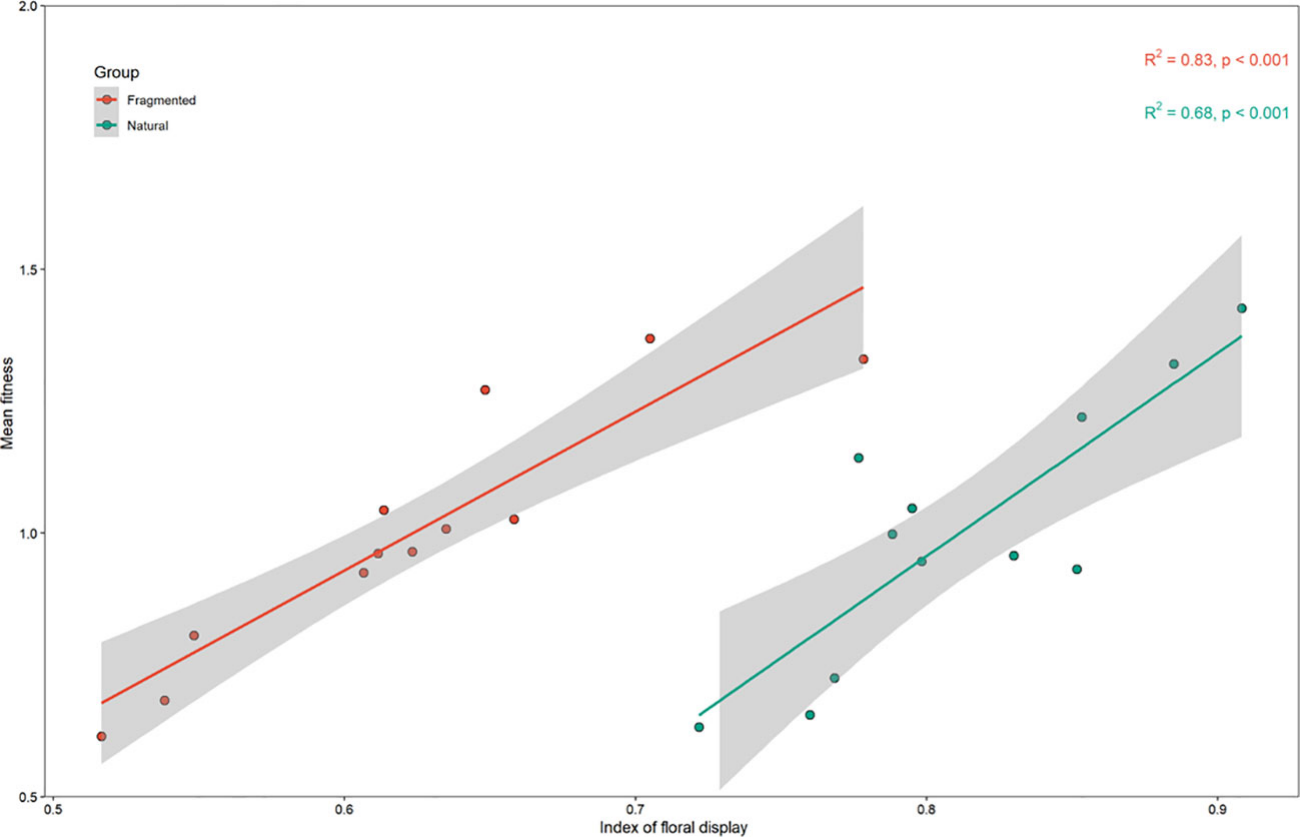
Mean female fitness was significantly related to the index of floral display.

The number of open flowers, corolla size, and keel petal length for *C. korshinskii* are shown in **Table 1**. Our results show that both habitats had similar corolla sizes and keel petal lengths, but the number of open flowers was significantly greater in the natural habitat than in the fragmented habitat (P < 0.05; **Table 1**). Female fitness was significantly correlated with the floral display index (R² = 0.68, Natural; R² = 0.82, Fragmented; **Figure 3**). Plants with a higher floral display index had greater seed production and seed mass.

**Table 1:** Floral display (Mean ± SD) for natural plants (N) and fragmented plants (F) in the *C. korshinskii*.

Floral Display	Natural	Fragmented	*P*
No. open flowers	29.0 ± 5.0	20.2 ± 3.9	< 0.05
Corolla size (cm^2^)	3.2 ± 0.9	2.8 ± 0.5	> 0.05
Keel petal length (mm)	17.2 ± 3.2	15.4 ± 4.2	> 0.05

### Pollen limitation

**Figure 4:**
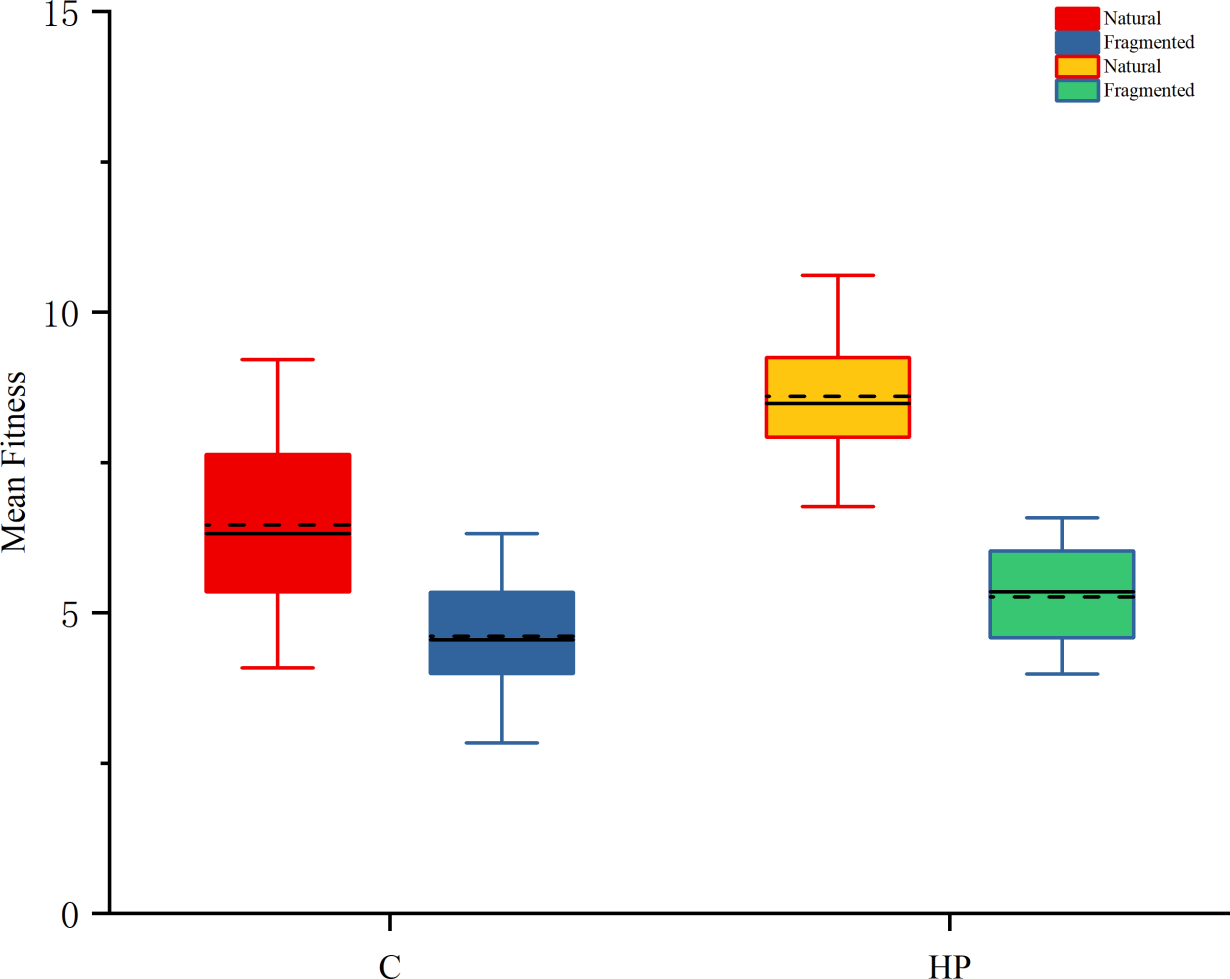
In natural and fragmented habitats, the mean fitness of *C. korshinskii* under different treatments (C and HP). C, open pollination treatment; HP, hand pollination treatment.

Female fitness was significantly higher in hand-pollinated plants compared to open-pollinated plants in both habitats (P < 0.05; **Table 2**). Our results suggest that pollen supplementation significantly increased mean fitness in both natural and fragmented habitats (**Figure 4**). Additionally, supplemental pollen increased female fitness by 32%, and the index of pollen limitation (I_PL_) was 0.24 in the natural habitat. Our findings indicate that this species experiences severe pollen limitation. For open-pollinated plants, the mean fitness was 6.5 in the natural habitat and 4.6 in the fragmented habitat. Plants in the natural habitat had significantly higher mean female fitness than those in the fragmented habitat (Habitat effect, *df* = 1, *P* < 0.001; **Table 3**).

**Table 2:** Mean female fitness (Mean ± SD) for open-pollinated plants (C) and hand-pollinated plants (HP) in different habitats.

Habitats	C	HP	*P*
Natural	6.5 ± 1.6	8.6 ± 1.1	< 0.05
Fragmented	4.6 ± 1.1	5.3 ± 0.8	< 0.05

**Table 3:** Impact of treatments (C and HP) and different habitats (Natural and Fragmented) on mean female fitness of C. *korshinskii*.

Traits	*df*	F	*P*
Treatment	1	11.050	< 0.05
Habitat	1	55.674	< 0.001
Treatment * Habitat	1	4.968	< 0.05

C, open pollination treatment; HP, hand pollination treatment.

### Natural selection gradients for floral display

**Figure 5:**
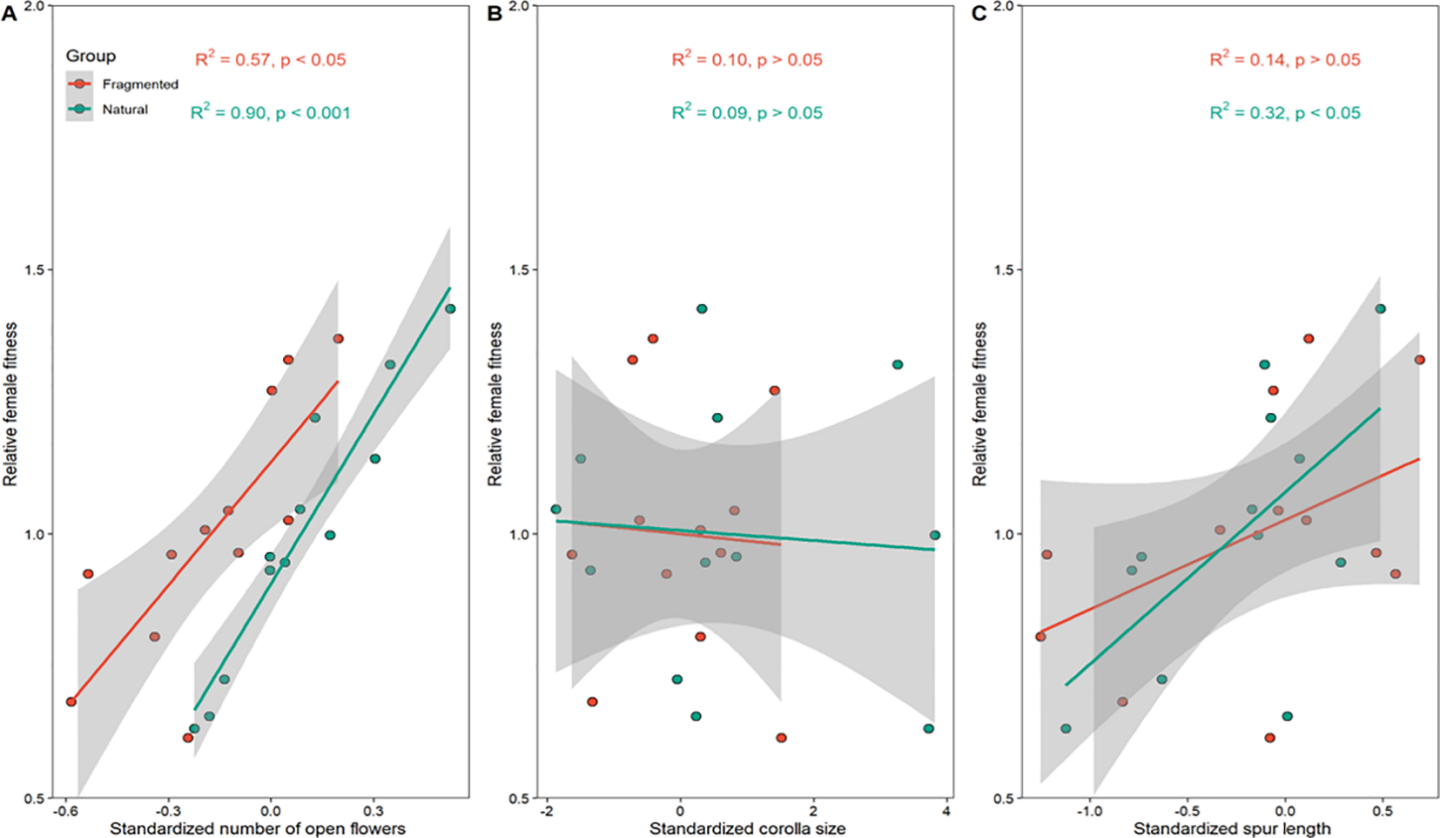
Standardized linear phenotypic selection gradients for **(A)** number of open flowers, **(B)** corolla size, and **(C)** keel petal length within the natural and fragmented habitats.

In *C. korshinskii*, directional selection favored plants with more open flowers and longer keel petals (**Figure 5A, C**). Additionally, corolla size was not significantly associated with selection (**Figure 5B**). Our findings suggest that the number of open flowers and keel petal length underwent significant positive directional selection (**Table 4**).

**Table 4:** Phenotypic linear selection gradients for plants in natural habitat (β_N_) and for plants in fragmented habitat (β_F_) in *C. korshinskii*.

Display	β_N_	β_F_	*P*_Display_	*P*_Display × Habitat_
No. open flowers	0.24	0.18	< 0.001	< 0.05
Corolla size	-0.02	-0.01	> 0.05	> 0.05
Keel petal length	0.16	0.11	< 0.001	> 0.05

Our results show that natural selection differed between the natural (β_N_) and fragmented habitats (β_F_; **Table 4**). In the natural habitat, the directional selection for the number of open flowers (β_N_) was 0.24, while the selection gradient for open flowers in the fragmented habitat was lower (β_F_ = 0.18; **Table 4**). Moreover, the intensity of directional selection on the number of open flowers and keel petal length in natural habitats was significantly higher than that in fragmented habitats (P_Display_ < 0.001; **Table 4**). In both habitats, our results also showed that habitat type significantly affected linear selection gradients for the number of open flowers (habitat effect, P_Display × Habitat_ < 0.05; **Table 4**).

### Effect of pollinator visitation frequency on female fitness in fragmented habitats

During the observation period, *Apis mellifera* ligustica Spinola was the most frequently observed floral visitor, constituting 81% of the 120 recorded insect visits. Additionally, the list of occasional insect visitors is provided in **Supplementary Table S1**. We found that *C. korshinskii* flowers possess a tripping mechanism, with visiting pollinators acting as tripping agents. Pollinators use their bodies to touch the petals, causing the flowers to open earlier. Furthermore, the visitation time of *A. mellifera* coincided with the observation period.

**Figure 6:**
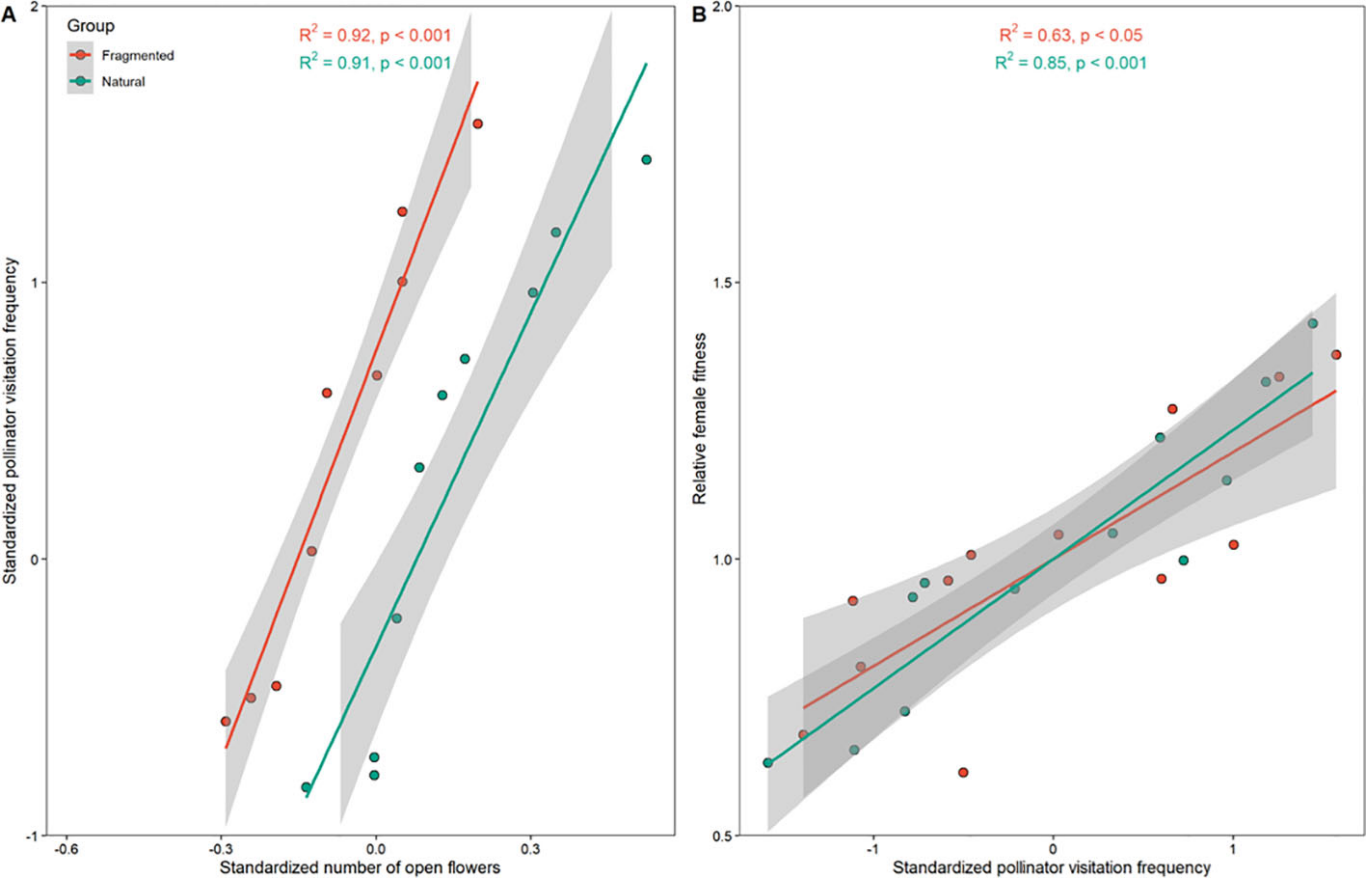
Relationships between number of open flowers (OF) and pollinator visitation frequency (V_F_) influence female fitness (FF). **(A)** Relationships between OF and V_F_, and **(B)** relationships between V_F_ and FF.

Pollinator visitation frequency significantly differed between the natural and fragmented habitats (P < 0.05). In both habitats, standardized pollinator visitation frequency was positively correlated with the number of open flowers (R² = 0.91, Natural; R² = 0.92, Fragmented; **Figure 6A**). In the natural habitat, seed production per selected flower was 4.6, and pollinator visitation frequency was 22.6. Our findings show that relative female fitness was significantly positively correlated with standardized pollinator visitation frequency (R² = 0.85, Natural; R² = 0.63, Fragmented; P < 0.01; **Figure 6B**).

**Figure 7:**
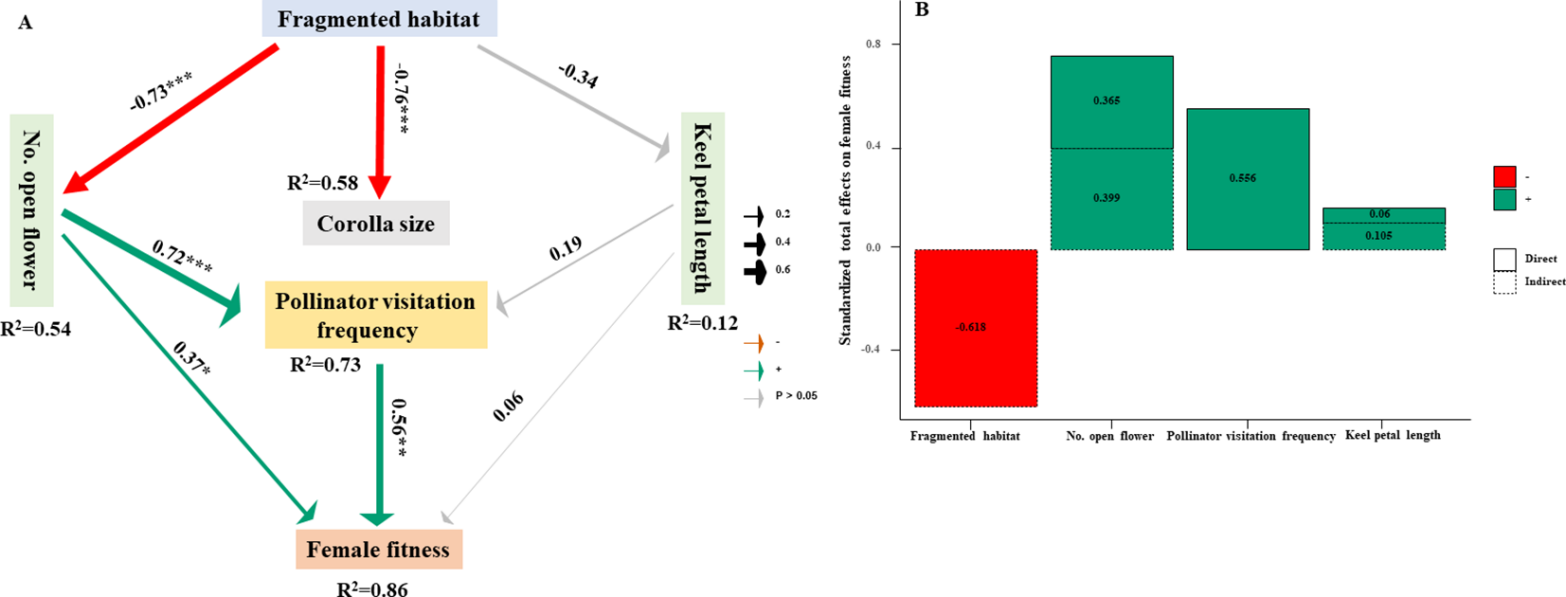
Structural equation modeling that depicts the direct and indirect impacts of fragmented habitat on female fitness. **(A)** Structural equation modeling revealing the impacts of the OF and V_F_ on female fitness (Fisher’s C= 13.65; *P* = 0.32; *df* = 12). The red and green arrows respectively show significant positive and negative effects (*P* < 0.05), while gray arrows indicate non-significant relationships. The values adjacent to the arrows represent standardized path coefficients. The width of the arrows corresponds to the strength of the path coefficients. R^2^ donates the proportion of variance explained. Significant levels for each predictor are denoted as *P < 0.05, **P < 0.01, ***P < 0.001. **(B)** Standardized total effects of all variables on female fitness derived from the SEM depicted above.

Although the fragmented habitat strongly affected the number of open flowers (OF) and corolla size, SEM results showed that the number of open flowers primarily influenced female fitness through its indirect effect on pollinator visitation frequency (OF → VF, covariance coefficient = 0.72) and direct effect on female fitness (OF → FF, covariance coefficient = 0.37) (**Figure 7A**). Our results indicate that keel petal length did not directly or significantly affect pollinator visitation frequency or female fitness. Furthermore, the fragmented habitat indirectly affected female fitness by cascading its effects on the number of open flowers, which in turn affected pollinator visitation frequency (VF → FF, covariance coefficient = 0.56). By calculating the total effects of each individual driver on female fitness, our findings suggest that the number of open flowers had the greatest positive and integrated effect on female fitness, followed by pollinator visitation frequency (**Figure 7B**). Additionally, the fragmented habitat had negative effects on female fitness.

## Discussion

### Study limitations

While this study advances our understanding of pollen limitation dynamics in fragmented habitats, three key methodological considerations warrant explicit discussion to contextualize its contributions. First, although we established correlations between pollinator visitation frequency and female fitness, our experimental design did not incorporate two critical components of pollination efficiency: (1) taxonomic identification of floral visitors, and (2) quantitative pollen transfer metrics (e.g., pollen load quantification on insect vectors or stigma pollen deposition counts). This gap constrains our ability to mechanistically link specific pollinator behaviors, such as interspecific variation in pollen carryover rates to observed differences in floral display selection pressures between habitat types.

Second, although pollen limitation assessments were conducted in both 2021 (pilot phase) and 2023, we strategically focused on 2023 data to minimize confounding effects of interannual environmental variability. While this approach aligns with our core objective of disentangling spatial drivers of pollen limitation, it necessarily excludes temporal dimension analyses. Future longitudinal studies spanning at least five reproductive cycles could elucidate whether the observed patterns represent evolutionarily stable selective regimes or ephemeral ecological responses.

### Pollen limitation and pollinators in fragmented habitats

Pollen quantity and quality may decrease due to insufficient pollen delivery or inefficient pollinator visits, leading to pollen limitation (Knight et al., 2005; Ashman et al., 2004). Many flowering plants experience severe pollen limitation due to limited pollen availability and unreliable pollinator visits (Lennartsson, 2002; Nayak and Davidar, 2010). The intensity of pollen limitation is also related to pollinator visitation efficiency, as pollination services are crucial for animal-pollinated plants (Liu et al., 2020). Previous studies indicate that fragmented habitats often reduce pollinator visitation efficiency and disrupt pollination processes (Newman et al., 2013; Chen et al., 2016). In this study, variation in floral displays often translates into differences in female fitness due to its effect on pollinator visitation frequency in fragmented habitats. Our findings show that *C. korshinskii* experiences more severe pollen limitation in fragmented habitats than in natural habitats, with supplemental hand pollination being the dominant factor influencing female fitness.

Our results demonstrate a positive impact of open flower number on female fitness within fragmented habitats (**Figure 7A**). This stems from enhanced nectar and pollen availability due to more open flowers, crucial for attracting pollinators. Consequently, optimal floral displays boost pollinator visitation and pollination efficiency (Vaughton and Ramsey, 2010). Moreover, our study indicates that floral resource abundance significantly influences pollinator visitation frequency and female fitness (**Figure 5**), highlighting the key role of open flower quantity in determining reproductive success for pollinator-dependent plants.

### Natural selection on floral display affects reproductive success in fragmented habitats

To understand how fragmented habitats influence floral display evolution, it is essential to quantify them as selective agents (Sletvold et al., 2017). An optimal floral display attracts pollinators and increases visitation frequency, with a positive correlation between flower density and pollinator visits (Ortíz et al., 2010). In fragmented habitats, plants with fewer flowers may experience directional selection, and floral display can reduce pollination efficiency (Kishore et al., 2012; Sletvold et al., 2017). In *C. korshinskii*, habitat fragmentation influences the selection pressure on the number of open flowers.

Plant–pollinator interactions drive floral diversity, and pollen transfer limits female reproductive success, suggesting that natural selection on floral display is widespread (Ashman and Morgan, 2004). Pollinators typically prefer more open flowers and longer keel petals, with the number of open flowers often setting the upper limit on seed production (Ashman et al., 2004; Gómez, 2003). Many studies show that the number of open flowers influences pollinator visitation and outcrossing rates, leading to higher seed production (Ortíz et al., 2010). In our study, 42% of plants in the natural habitat were strongly selected for more open flowers. In the fragmented habitat, 50% of plants were under directional selection for a greater number of open flowers. Shortening keel petal length significantly reduces female fitness, while longer keel petals enhance pollination efficiency (Sletvold et al., 2017; Gómez, 2003). Additionally, this species exhibits directional selection for longer keel petals, though habitat type does not significantly affect the linear selection gradient for keel petal length. Our study is the first to experimentally quantify the impact of natural selection on floral display in *Caragana* species across different habitats. This study enhances our understanding of how fragmented habitats shape floral display evolution in *C. korshinskii.*

Many studies have noted a challenge in quantifying natural selection through hand-pollination treatments, particularly in distinguishing the effects of display variation on pollen quality and quantity (Sletvold et al., 2017). In this study, however, the impact of the hand-pollination treatment on pollen quality was minimized. In *C. korshinskii*, self-pollination efficiency is low. Additionally, hand-pollinated plants received a mixture of pollinator-transferred and hand-supplemented pollen, with minimal differences in pollen quality. Therefore, natural selection on floral display reflects variation in pollen deposition quantity.

### Relationships between pollinator visitation and female fitness in fragmented habitats

Plants are immobile and rely on pollinators for pollen transfer, a critical strategy for sexual reproduction (Harder and Aizen, 2010). Pollinator selection of floral display plays a key role in pollen transfer and seed production (Karron and Mitchell, 2012; Gómez, 2003). Over the past two decades, the fragmentation of natural habitats due to urbanization and human disturbance has increased significantly (Delnevo et al., 2019). In fragmented habitats, the spatial distance between animal-pollinated plants increases, while pollinator visit time decreases, resulting in lower pollinator visitation frequency and increased genetic isolation of plants (Newman et al., 2013). Pollinator services primarily depend on visitation efficiency, which is considered a direct causal factor influencing female fitness (Knight et al., 2005; Herrera, 2020). Pollinator visits are positively correlated with flower resource density, and higher visitation frequency can enhance cross-pollen transport (Ortíz et al., 2010). Additionally, habitat fragments increase edge effects on potential pollinator visits, which may prevent or hinder pollinator movement between fragments. In this study, *Apis mellifera* was more common in natural habitats. Moreover, plants in natural habitats exhibited more open flowers than those in fragmented habitats, offering an explanation for the observed difference in female fitness.

Habitat fragmentation is a widespread change in terrestrial ecosystems that profoundly impacts plant-pollinator interactions (Potts et al., 2016). Plant reproductive success significantly affects the population viability and ecological stability of desert steppes (Chen et al., 2022). In desert steppes, pollinator abundance and diversity are declining due to human-related factors such as habitat fragmentation, climate change, and overgrazing (Potts et al., 2016). Habitat change affects biomass allocation to reproductive organs, leading to altered reproductive strategies (Revel et al., 2012). A suitable floral display can enhance pollination services and reproductive success (Sletvold et al., 2017). Previous studies suggest that floral display influences pollinator selection and is linked to habitat loss and climate change (Revel et al., 2012; Delnevo et al., 2019). Similar studies have shown that plants exhibit lower female fitness when pollinator visitation frequency decreases, with visitation linked to reproductive success (Cresswell, 1997; Suzuki, 2000; Jones and Agrawal, 2017). In our study, pollinator visitation frequency positively influenced female fitness, which is considered a key indicator of reproductive success. Most importantly, we found that the number of open flowers, rather than corolla size or keel petal length, was the primary driver influencing the relationship between pollinator visitation frequency and female fitness, with fragmented habitat affecting female fitness through its cascading effects on the number of open flowers. Our study provides strong evidence that the effects of habitat fragmentation cascade through multiple levels, influencing various factors. Most previous studies on floral display-reproduction relationships focus on a single level, whereas this study advances our understanding of the overall effects of habitat fragmentation on female fitness from multiple perspectives. Therefore, this study demonstrates that fragmented habitats affect reproductive success through natural selection on the number of open flowers and pollinator visitation frequency in desert steppes.

## Conclusions

We experimentally quantified how fragmented habitats affect floral display and natural selection on pollinator visitation. In *C. korshinskii*, 42% of plants in the natural habitat were strongly selected for more open flowers (β_N_ = 0.24), and 50% of plants in fragmented habitats showed directional selection for greater numbers of open flowers (β_F_ = 0.18). We conclude that the number of open flowers is the primary selective factor, with fragmented habitats significantly influencing the linear selection gradients for this trait. Additionally, the number of open flowers directly and significantly impacts pollinator visitation frequency and female fitness. Our work provides the first evidence that changes in the number of open flowers in fragmented habitats are a primary driver of floral display-reproduction relationships. This study highlights the need to investigate the interactions between floral display and pollinator visitation in fragmented habitats to better manage pollen limitation and reproductive success in dominant Fabaceae species.

## Data Availability

The raw data supporting the conclusions of this article will be made available by the authors, without undue reservation.

## References

[B1] Ashman T. L. Knight T. M. Steets J. A. Amarasekare P. Burd M. Campbell D. R.. (2004). Pollen limitation of plant reproduction: ecological and evolutionary causes and consequences. Ecology 85, 2408–2421. doi: 10.1890/03-8024

[B2] Ashman T. L. Morgan M. T. (2004). Explaining phenotypic selection on plant attractive characters: male function, gender balance or ecological context? Proc. R. Soc Lon. B 271, 553–559. doi: 10.1098/rspb.2003.2642 PMC1691631 15156911

[B3] Asikainen E. Mutikainen P. (2005). Pollen and resource limitation in a gynodioecious species. Am. J. Bot. 92, 487–494. doi: 10.3732/ajb.92.3.487 21652426

[B4] Chen M. Zhao X. (2019). Impact of floral characters, pollen limitation, and pollinator visitation on pollination success in different populations of *Caragana korshinskii* Kom. Sci. Rep. 9, 9741. doi: 10.1038/s41598-019-46271-z 31278340 PMC6611805

[B5] Chen M. Zhao X. Y. Yue P. Guo X. X. Qiao J. J. Li X. Y. (2022). Effect of grazing disturbance on floral display, pollen limitation and plant pollination efficiency in the desert steppe. BMC Plant Biol. 22, 514. doi: 10.1186/s12870-022-03899-w 36329386 PMC9635133

[B6] Chen M. Zhao X. Y. Zuo X. A. Mao W. Hao Q. Zhu Y. C. (2016). Effects of habitat disturbance on the pollination system of *Ammopiptanthus mongolicus* (Maxim) Cheng f. at the landscape-level in an arid region of Northwest China. J. Plant Res. 129, 435–447. doi: 10.1007/s10265-015-0779-7 26780064

[B7] Chen M. Zuo X. A. (2019). Effect of pollen limitation and pollinator visitation on pollination success of Haloxylon ammodendron (C. A. Mey.) Bunge in fragmented habitats. Front. Plant Sci. 10. doi: 10.3389/fpls.2019.00327 PMC6448003 30984212

[B8] Chen M. Zuo X. A. Zhao X. Y. (2020). Comparative floral characters, pollinator limitation and pollination success in different habitats of *Caragana microphylla* Lam. Front. Ecol. Evol. 8. doi: 10.3389/fevo.2020.00170

[B9] Corbett S. A. (2003). Nectar sugar content: estimating standing crop and secretion rate in the field. Apidologie. 34, 1–10. doi: 10.1051/apido:00049

[B10] Cresswell J. E. (1997). Spatial heterogeneity, pollinator behaviour and pollinator-mediated gene flow: Bumblebee movements in variously aggregated rows of oil-seed rape. Oikos 78, 546–556. doi: 10.2307/3545616

[B11] Delnevo N. Van Etten E. J. Byrne M. Stock W. D. (2019). Floral display and habitat fragmentation: Effects on the reproductive success of the threatened mass-flowering *Conospermum undulatum* (Proteaceae). Ecol. Evol. 9, 11494–11503. doi: 10.1002/ece3.v9.19 31641488 PMC6802041

[B12] Fernández J. D. Bosch J. Nieto-Ariza B. Gómez J. M. (2012). Pollen limitation in a narrow endemic plant: geographical variation and driving factors. Oecologia 170, 421–431. doi: 10.1007/s00442-012-2312-1 22492167

[B13] Gigord L. D. B. Macnair M. R. Smithson A. (2001). Negative frequency-dependent selection maintains a dramatic flower color polymorphism in the rewardless orchid *Dactylorhiza sambucina* (L.) Soo. Proc. Natl. Acad. Sci. U.S.A. 98, 6253–6255. doi: 10.1073/pnas.111162598 11353863 PMC33454

[B14] Gómez J. M. (2003). Herbivory reduces the strength of pollinator-mediated selection in the Mediterranean herb *Erysimum mediohispanicum*: Consequences for plant specialization. Am. Nat. 162, 242–256. doi: 10.1086/376574 12858267

[B15] Gómez J. M. Abdelaziz M. Lorite J. Munõz-Pajares A. J. Perfectti F. (2010). Changes in pollinator fauna cause spatial variation in pollen limitation. J. Ecol. 98, 1243–1252. doi: 10.1111/j.1365-2745.2010.01691.x

[B16] Guo Z. Chang H. T. Liu R. T. (2022). Response of soil microarthropod community to seasonal changes in urat desert steppe, inner Mongolia. Front. Environ. Sci. 10. doi: 10.3389/fenvs.2022.893913

[B17] Hadley A. S. Betts M. G. (2012). The effects of landscape fragmentation on pollination dynamics: absence of evidence not evidence of absence. Biol. Rev. 87, 526–544. doi: 10.1111/j.1469-185X.2011.00205.x 22098594

[B18] Harder L. D. Aizen M. A. (2010). Floral adaptation and diversification under pollen limitation. Philos. T. R. Soc B 365, 529–543. doi: 10.1098/rstb.2009.0226 PMC2838256 20047878

[B19] Herrera C. M. (2020). Flower traits, habitat, and phylogeny as predictors of pollinator service: A plant community perspective. Ecol. Monogr. 90, 1402. doi: 10.1002/ecm.1402

[B20] Hu Z. K. Delgado-Baquerizo M. Fanin N. Chen X. Y. Zhou Y. Du G. Z.. (2024). Nutrient-induced acidification modulates soil biodiversity-function relationships. Nat. Commun. 15, 2858. doi: 10.1038/s41467-024-47323-3 38570522 PMC10991381

[B21] Ishii J. Kadono Y. (2002). Factors influencing seed production of Phragmites australis. Aquat. Bot. 72, 129–141. doi: 10.1016/S0304-3770(01)00218-2

[B22] Jones P. L. Agrawal A. A. (2017). Learning in insect pollinators and herbivores. Annu. Rev. Entomol. 62, 53–71. doi: 10.1146/annurev-ento-031616-034903 27813668

[B23] Karron J. D. Mitchell R. J. (2012). Effects of floral display size on male and female reproductive success in *Mimulus ringens* . Ann. Bot-London. 109, 563–570. doi: 10.1093/aob/mcr193 PMC3278290 21880660

[B24] Kishore K. Shukla A. K. Babu N. Sarangi D. N. Patanayak S. (2012). Pollination biology of *Annona squamosa* L. (Annonaceae): Evidence for pollination syndrome. Sci. Hortic-Amsterdam. 144, 212–217. doi: 10.1016/j.scienta.2012.07.004

[B25] Knight T. M. Steets J. A. Vamosi J. C. Mazer S. J. Burd M. Campbell D. R.. (2005). Pollen limitation of plant reproduction: pattern and process. Annu. Rev. Ecol. Evol. Syst. 36, 467–497. doi: 10.1146/annurev.ecolsys.36.102403.115320

[B26] Lefcheck J. S. (2016). piecewiseSEM: Piecewise structural equation modelling in R for ecology, evolution, and systematics. Methods Ecol. Evol. 7, 573–579. doi: 10.1111/mee3.2016.7.issue-5

[B27] Lennartsson T. (2002). Extinction thresholds and disrupted plant-pollinator interactions in fragmented plant populations. Ecology 83, 3060–3072.

[B28] Liu R. R. Chen D. L. Luo S. D. Xu S. J. Xu H. L. Shi X. Y.. (2020). Quantifying pollination efficiency of flower-visiting insects and its application in estimating pollination services for common buckwheat. Agric. Ecosystems Environment. 301, 107011. doi: 10.1016/j.agee.2020.107011

[B29] Nayak K. G. Davidar P. (2010). Pollinator limitation and the effect of breeding systems on plant reproduction in forest fragments. Acta Oecol. 36, 191–196. doi: 10.1016/j.actao.2009.12.004

[B30] Newman B. J. Ladd P. Brundrett M. Dixon W. K. (2013). Effects of habitat fragmentation on plant reproductive success and population viability at the landscape and habitat scale. Biol. Conserv. 159, 16–23. doi: 10.1016/j.biocon.2012.10.009

[B31] Ortíz F. E. Stoner K. Pérez-Negrón E. Casas A. (2010). Pollination biology of Myrtillocactus schenckii (Cactaceae) in wild and managed populations of the Tehuacán Valley, México. J. Arid. Environ. 74, 897–904. doi: 10.1016/j.jaridenv.2010.01.009

[B32] Potts S. G. Imperatriz-Fonseca V. Ngo H. T. Aizen M. A. Biesmeijer J. C. Breeze T. D.. (2016). Safeguarding pollinators and their values to human well-being. Nature 540, 220–229. doi: 10.1038/nature20588 27894123

[B33] Revel N. Alvarez N. Gibernau M. Anahí E. (2012). Investigating the relationship between pollination strategies and the size-advantage model in zoophilous plants using the reproductive biology of Arum cylindraceum and other European Arum species as case studies. Arthropod-Plant Interact. 6, 35–44. doi: 10.1007/s11829-011-9164-1

[B34] Sletvold N. Ågren J. (2010). Pollinator-mediated selection on floral display and spur length in the orchid Gymnadenia conopsea. Int. J. Plant Sci. 171, 999–1009. doi: 10.1086/656597 26183369

[B35] Sletvold N. Grindeland J. M. Ågren J. (2010). Pollinator-mediated selection on floral display, spur length and flowering phenology in the deceptive orchid *Dactylorhiza lapponica* . New Phytol. 188, 385–392. doi: 10.1111/j.1469-8137.2010.03296.x 20497348

[B36] Sletvold N. Tye M. Ågren J. (2017). Resource- and pollinator-mediated selection on floral traits. Funct. Ecol. 31, 135–141. doi: 10.1111/fec.2017.31.issue-1

[B37] Steffan-Dewenter I. Klein A. M. Gaebele V. Alfert T. Tscharntke T. (2006). “Bee diversity and plant-pollinator interactions in fragmented landscapes,” in Plant-pollinator interactions: from specialization to generalization. Eds. Waser N. M. Ollerton J. (Chicago: University of Chicago Press), 387–407.

[B38] Sun H. Q. Huang B. Q. Yu X. H. Tian C. B. Peng Q. X. An D. J. (2018). Pollen limitation, reproductive success and flowering frequency in single-flowered plants. J. Ecol. 106, 19–20. doi: 10.1111/1365-2745.12834

[B39] Suzuki N. (2000). Pollinator limitation and resource limitation of seed production in the Scotch broom, Cytisus scoparius (Leguminosae). Plant Spec. Biol. 15, 187–193. doi: 10.1046/j.1442-1984.2000.00038.x

[B40] Vaughton G. Ramsey M. (2010). Floral emasculation reveals pollen quality limitation of seed output in Bulbine bulbosa (Asphodelaceae). Am. J. Bot. 97, 174–178. doi: 10.3732/ajb.0900183 21622377

[B41] Villagra P. E. Defossé G. E. Del Valle H. F. Tabeni S. Rostagno M. Cesca E.. (2009). Land use and disturbance effects on the dynamics of natural ecosystems of the Monte Desert: implications for their management. J. Arid Environ. 73, 202–211. doi: 10.1016/j.jaridenv.2008.08.002

[B42] Wang G. H. Chen Z. X. Yang X. L. Cai G. J. Shen Y. Y. (2022). Effect of simulated precipitation regimes on sap flow and water use efficiency for xerophytic *Caragana korshinskii* . Ecol. Indic. 143, 109309. doi: 10.1016/j.ecolind.2022.109309

[B43] Yao Y. Shao M. Fu X. Wang X. Wei X. (2019). Effects of shrubs on soil nutrients and enzymatic activities over a 0–100 cm soil profile in the desert-loess transition zone. Catena 174, 362–370. doi: 10.1016/j.catena.2018.11.031

[B44] Zhong M. X. Song J. Zhou Z. X. Ru J. Y. Zheng M. M. Li Y.. (2019). Asymmetric responses of plant community structure and composition to precipitation variabilities in a semi−arid steppe. Oecologia 191, 697–708. doi: 10.1007/s00442-019-04520-y 31578614

