# Supplementary material for: Impact of fragmented habitats on reproductive success in dominant shrubs: natural selection on floral display and pollinator visitation
Source: Front Plant Sci. 2025 Jun 5;16:1522871. doi: 10.3389/fpls.2025.1522871 (PMC12178238; doi:10.3389/fpls.2025.1522871)
Supplement: Supplementary file 1 [file Table1.docx]

**Table S1.** The list of occasional pollinators in *C. korshinskii*.

|  | **Order** | **Family** | **Genus** |
| --- | --- | --- | --- |
| *Episyrphus balteatus* | Diptera | Syrphidae | Episyrphus |
| *Megachile* (Chalicodoma) *desertorum* Morawitz | Hymenoptera | Megachilidae | Megachile |
